# Implementation of the TaperGuard™ endotracheal tube in an unselected surgical population to reduce postoperative pneumonia

**DOI:** 10.1186/s12871-020-01117-4

**Published:** 2020-08-24

**Authors:** Ross P. Martini, N. David Yanez, Miriam M. Treggiari, Praveen Tekkali, Cobin Soelberg, Michael F. Aziz

**Affiliations:** 1 Department of Anesthesiology and Perioperative Medicine, Oregon Health & Science University, Oregon, USA; 2 Department of Anesthesiology, Yale University, 333 Cedar Street, TMP-3, New Haven, CT US 06510 USA

**Keywords:** Nosocomial infection, Hospital acquired pneumonia, Ventilator associated pneumonia, Aspiration, Postoperative complications

## Abstract

**Background:**

Endotracheal tube (ETT) designs to decrease the risk of ventilator associated pneumonia (VAP) include supraglottic suctioning, and/or modifications of the cuff shape. The TaperGuard™ ETT has a tapered, polyvinylchloride cuff designed to reduce microaspiration around channels that form with a standard barrel-shaped cuff. We compared risk of postoperative pneumonia using the TaperGuard™ ETT and the standard ETT in surgical patients requiring general anesthesia with endotracheal intubation.

**Methods:**

We used an interrupted time-series design to compare endotracheal intubation using the TaperGuard™ ETT (intervention cohort), and a historic cohort using the standard ETT (baseline cohort), among surgical patients requiring hospital admission. We compared the incidence of postoperative pneumonia in the intervention and baseline cohorts. Data were collected from the electronic health record and linked to patient-level data from National Surgical Quality Improvement Project. Additionally, we performed secondary analyses in a subgroup of patients at high risk of postoperative pneumonia.

**Results:**

15,388 subjects were included; 6351 in the intervention cohort and 9037 in the baseline cohort. There was no significant difference in the incidence of postoperative pneumonia between the intervention cohort (1.62%) and the baseline cohort (1.79%). The unadjusted odds ratio (OR) of postoperative pneumonia was 0.90 (95% CI: 0.70, 1.16; *p* = 0.423) and the OR adjusted for patient characteristics and potential confounders was 0.90 (95% CI: 0.69, 1.19; *p* = 0.469), comparing the intervention and baseline cohorts. There was no a priori selected subgroup of patients for whom the use of the TaperGuard™ ETT was associated with decreased odds of postoperative pneumonia relative to the standard ETT. Hospital mortality was higher in the intervention cohort (1.5%) compared with the baseline cohort (1.0%; OR 1.46, 95% CI: 1.09, 1.95; *p* = 0.010).

**Conclusions:**

The broad implementation of the use of the TaperGuard™ ETT for intubation of surgical patients was not associated with a reduction in the risk of postoperative pneumonia. In the setting of a low underlying postoperative pneumonia risk and the use of recommended preventative VAP bundles, further risk reduction may not be achievable by simply modifying the ETT cuff design in unselected or high-risk populations undergoing inpatient surgery.

**Trial registration:**

ClinicalTrials.gov, ID NCT02450929.

## Background

In high-risk surgical patient populations, the incidence of postoperative pneumonia has been reported to be as high as 21% [1–3]. Recent national efforts to decrease healthcare associated infections have resulted in the development of a bundle of interventions that have effectively decreased the incidence of VAP in high-risk patient populations [4].

Strategies to minimize microaspiration of supraglottic secretions include modification of the endotracheal tube (ETT) configuration and the utilization of supraglottic suctioning [5–7]. The TaperGuard™ ETT (Covidien, Boulder, CO) is designed to prevent microaspiration around channels that otherwise form with a barrel-shaped cuff (standard ETT) and may thereby reduce the incidence of VAP. This device has undergone multiple independent and manufacturer sponsored laboratory and clinical trials. Laboratory evaluations have demonstrated decreased passage of fluid or dye around the ETT compared with conventional barrel-shaped cuffs, but not in all experimental conditions [8–14].

Clinical evaluations in relatively small studies of unselected patient populations have failed to demonstrate a decrease in the incidence of postoperative pneumonia [15, 16]. Because the incidence of postoperative pneumonia in an otherwise unselected patient population is much lower than in those with prolonged ventilation or other risk factors, it is possible that prior trials were too small to detect a difference, or that selection of subgroups were suboptimal.

As part of a quality improvement initiative, our institution implemented a change of ETTs from the standard barrel-shaped design to the TaperGuard™ ETT for all surgical patients. The present study compares the incidence of postoperative pneumonia, before and after the implementation of the TaperGuard™ ETT in a large, unselected inpatient population undergoing surgery with general anesthesia and in several high-risk subgroups, to determine the efficacy of this device in reducing postoperative pneumonia.

## Methods

### Study design

This cohort study was conducted in the setting of the implementation of a perioperative quality improvement initiative within the Department of Anesthesiology and Perioperative Medicine at Oregon Health & Science University (OHSU) Hospital. On December 1, 2012, OHSU instituted a practice change to transition from ETTs with a barrel-shaped cuff design to the TaperGuard™ ETT for all surgical patients. We used an interrupted time-series to compare two cohorts of patients undergoing inpatient surgery with general anesthesia and the placement of an ETT, during a baseline period with the use of standard ETT and an intervention period with the use of the TaperGuard™ ETT. The baseline cohort included patients who had surgery between April 1, 2011 and November 30, 2012; the intervention cohort included patients who had surgery between December 1, 2012 and February 15, 2014. The collection and review of clinical information for this study was approved by the OHSU institutional review board, which waived the need for informed consent. The study was registered on clinicaltrials.gov as NCT02450929. This manuscript adheres to the applicable SQUIRE 2.0 guidelines.

During the intervention period, there were no active institutional changes to address postoperative pneumonia. Patients admitted to the ICU received a uniform pneumonia prevention bundle including oral care, head of bed elevation, daily sedation interruptions with spontaneous breathing trials, and appropriate stress-ulcer prophylaxis. There were no other institutional changes to operating room management during the two study periods, including default ventilator settings, aspiration prevention techniques, and oral care.

### Patient population

All elective and emergency surgical patients undergoing procedures in the operating room that required endotracheal intubation followed by postoperative hospitalization were included in the study. We excluded patients younger than 18 years of age. For patients undergoing multiple surgeries during a single hospitalization, only the first surgical event of the hospitalization was described.

### Outcomes and data collection

The primary outcome was postoperative pneumonia during the hospitalization, identified based on hospital discharge ICD-9 codes for bacterial and fungal pneumonia and included the following specific codes: 481.00–486.99 (pneumonia) and 997.31 (VAP). Centricity (General Electric, Fairfield, CT) and EPIC (Verona, WI) anesthesia information management systems were queried for clinical data. An internal OHSU perioperative patient database that included a cohort of these patients was also used to collect baseline demographics, characteristics of anesthetic and surgical perioperative care, and postoperative variables.

### Statistical analysis

Summary statistics (means and standard deviations for quantitative characteristics and frequencies and percentages for categorical factors) were estimated for patients’ demographic characteristics (i.e., age, race, gender, body mass index), perioperative factors (i.e., American Society of Anesthesiologists [ASA] physical status classification, procedural classifications) and potential confounding factors (i.e., tidal volume, rapid sequence intubation, use of non-depolarizing neuromuscular blockade, positive end-expiratory pressure [PEEP]). We first compared the characteristics between the baseline and intervention cohorts using two-sample unequal variance t-test for the quantitative characteristics or chi-squared test statistics for the categorical ones. We also presented ETT cohort (unadjusted) summary statistics for the primary outcomes: (a) VAP, using a chi-square test, (b) duration of mechanical ventilation (min), and (c) hospital length of stay (days). The latter two comparisons were made using an unequal variance t-test. We then formally evaluated whether there were differences in these outcomes by adjusting (controlling) for potential confounding factors. For the postoperative pneumonia outcome and hospital mortality outcome, we performed multivariable logistic regression to test whether the odds of postoperative pneumonia or mortality in the intervention cohort were different than the odds of postoperative pneumonia or mortality in the baseline cohort. For the two quantitative secondary outcomes, hospital length of stay (LOS) and duration of mechanical ventilation (min), we performed multivariable linear regression using robust (sandwich) estimated standard errors to remedy possible violation of the model variance assumption. We tested for differences in the two cohorts using standard (adjusted) pairwise comparison tests.

Finally, we explored whether possible effects between postoperative pneumonia and the two cohorts were modified by the following potential effect modifiers: diabetes, hypertension, COPD, tobacco abuse, ischemic heart disease, GERD, heart failure, obesity, and intraoperative use of nondepolarizing neuromuscular blockade. These analyses were performed using multivariable logistic regression models. Separate models were fitted for each of the potential effect modifiers, similar to the primary adjusted analysis for postoperative pneumonia, but with the inclusion of one additional term for the effect modifier and its interaction term with the cohort predictor. The a priori selected confounding factors included in all adjusted analyses were ASA status (five categories), tidal Volume (ml/kg), Caucasian race (yes/no), age (in years), male gender (yes/no), and PEEP (=0 or > 0). All hypothesis tests, associated *p*-values and confidence intervals were two-sided. The statistical analyses were performed using Stata (ver. 15.1) and R (ver. 3.3.3) statistical packages.

## Results

### Demographics

**Fig. 1:**
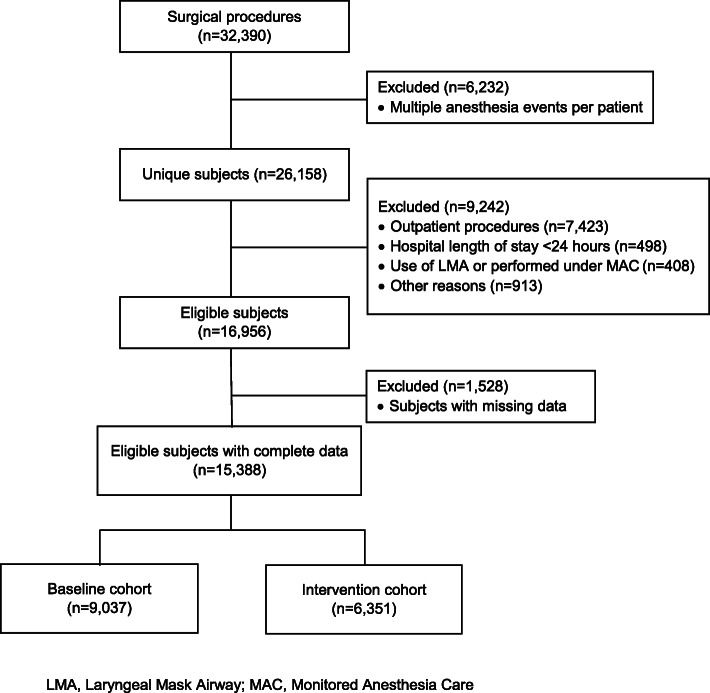
Study flowchart

The study flowchart is shown in Fig. 1. During the two study periods, 16,956 patients were potentially eligible. Of these patients, a total of 15,388 (91%) had complete data, with 9037 patients in the baseline cohort and 6351 in the intervention cohort (Table 1). Mean age (*p* <  0.001), Caucasian race (*p* = 0.004), ASA class (*p* <  0.001) and surgical category (*p* = 0.001) were significantly different among cohorts. However, the differences between these cohorts were not clinically relevant. Gender and mean body mass index were not different between the baseline and intervention cohorts. The frequency distribution of surgical procedures was also similar between the two cohorts in terms of clinical significance, even though they differed statistically (*p* = 0.001). The significant differences were due to the high degree of precision of the estimates because of the large sample size.

**Table 1:** Demographic and intraoperative characteristics of patients intubated with standard barrel cuff endotracheal tube (baseline) and the TaperGuard™ endotracheal tube (intervention). Data are expressed as mean (standard deviation) unless otherwise specified

Characteristics	Baseline (***n*** = 9037)	Intervention (***n*** = 6351)	***p*** value
Age, years	55.2 ± 16.6	56.3 ± 16.5	< 0.001
Male, n (%)	4515 (50.0)	3186 (50.2)	0. 803
Caucasian, n (%)	8042 (93.0)	5748 (91.7)	0.004
Body Mass Index, kg/m^2^	29.9 ± 7.9	30.0 ± 8.1	0.222
ASA Class, n (%)			< 0.001
1	505 (5.7)	336 (5.3)	
2	3547 (40.0)	2421 (38.5)	
3	3949 (44.5)	2780 (44.2)	
4	845 (9.5)	739 (11.8)	
5	21 (0.2)	15 (0.2)	
Surgical procedure, n (%)			0.001
Open abdominal	1811 (20.0)	1339 (21.1)	
Laparoscopic abdominal	1536 (17.0)	997 (15.7)	
Orthopedic	1403 (15.5)	936 (14.7)	
Neurosurgery	778 (8.6)	551 (8.7)	
Spine	1155 (12.8)	801 (12.6)	
Breast/soft tissue	693 (7.7)	458 (7.2)	
Thoracic	190 (2.1)	132 (2.1)	
Vascular	337 (3.7)	240 (3.8)	
Cardiac	519 (5.7)	451 (7.1)	
Otolaryngology	443 (4.9)	318 (5.0)	
Out of OR locations	139 (1.5)	120 (1.9)	
Ophthalmology	33 (0.4)	8 (0.1)	
Rapid sequence intubation, n (%)	258 (2.9)	173 (2.7)	0.628
Median tidal volume, mL/kg	8.2 (1.6)	7.9 (1.4)	< 0.001
Median tidal volume, mL	514.5 (80.7)	498.0 (73.4)	< 0.001
Mean tidal volume, mL	487.6 (76.0)	473.7 (69.9)	< 0.001
PEEP, n (%)	8577 (95.2)	6172 (97.4)	< 0.001
Non-depolarizing NMB, n (%)	7630 (84.4)	5389 (84.9)	0.475

*ASA* American Society of Anesthesiologists, *PEEP* positive end expiratory pressure, *NMB* neuromuscular blockade

### Intraoperative characteristics

Intraoperative characteristics are also shown in Table 1, stratified by ETT cohort. The use of rapid sequence intubation and the utilization of a non-depolarizing neuromuscular blockade were not significantly different in the two cohorts. The intervention cohort had a significantly lower median tidal volume of 7.9 ± 1.4 mL/kg compared to the baseline cohort (8.2 ± 1.6 mL/kg, *p* <  0.001), as well as a higher utilization of PEEP intraoperatively (intervention, 97.4% vs. baseline 95.2%, *p* <  0.001). The mean procedure duration was similar between the cohorts (mean difference = 2.0 min, 95% CI: − 6.0, 2.0; *p* = 0.333).

### Study endpoints

Table 2 provides unadjusted and adjusted estimates for the study primary and secondary endpoints. The unadjusted estimates of the incidence of postoperative pneumonia were 1.62% in the intervention cohort and 1.79% in the baseline cohort (OR 0.9, 95% CI: 0.70, 1.16; *p* = 0.423). The estimated mean duration of mechanical ventilation was also not different between the two cohorts (mean difference = 2.0 min, 95% CI: − 2.0, 6.0; *p* = 0.333). Hospital length of stay was 0.6 days longer for the Intervention cohort (95% CI: 0.3, 0.9; *p* <  0.001). The associations remained unchanged after adjustment for potential confounders. The adjusted difference in the estimated hospital length of stay was slightly attenuated with a mean difference of 0.5 days longer for the intervention cohort (95% CI: 0.2, 0.8; *p* <  0.001) compared to the baseline cohort. Hospital mortality was significantly higher in the intervention group (1.5%) compared to the baseline cohort (1.0%; OR 1.46, 95% CI: 1.09, 1.95; *p* = 0.010), which remained unchanged after adjustment for potential confounders.

**Table 2:** Study primary and secondary endpoints among baseline (standard ETT) and intervention (TaperGuard™ ETT) cohorts. Data are expressed as mean (standard deviation) unless otherwise specified

Characteristics	Baseline (***n*** = 9037)	Intervention (***n*** = 6351)	Baseline vs. Intervention (95% Confidence Interval)	***p*** value
Ventilator associated pneumonia^a^, n (%)	162 (1.79)	103 (1.62)		
Unadjusted OR			0.90 (0.70, 1.16)	0.423
Adjusted OR ^c^			0.90 (0.69, 1.19)	0.469
Duration of mechanical ventilation^b^, min	208.9 (112.6)	210.9 (112.2)		
Unadjusted difference			2.0 (−2.0, 6.0)	0.333
Adjusted difference^c^			1.6 (−2.7, 5.9)	0.476
Hospital length of stay, days	6.2 (7.9)	6.8 (9.1)		
Unadjusted difference			0.6 (0.3, 0.9)	< 0.001
Adjusted difference^c^			0.5 (0.2, 0.8)	< 0.001
Hospital mortality, n (%)	92 (1.0)	94 (1.5)	1.46 (1.09, 1.95)	0.010
Unadjusted OR			1.40 (1.02, 1.93)	0.039
Adjusted OR^c^				

*ASA* American Society of Anesthesiologists, *NMB* neuromuscular blockade, *PEEP* positive end expiratory pressure

^a^Summaries are odds ratios (OR) for VAP: Intervention compared with baseline

^b^Summaries are mean differences (Diff): Intervention compared with baseline

^c^Adjustment variables are: ASA status (1–5), Tidal Volume (ml/kg), Caucasian race (yes, no), age (years), male gender (yes, no), PEEP (0 and > 0)

**Fig. 2:**
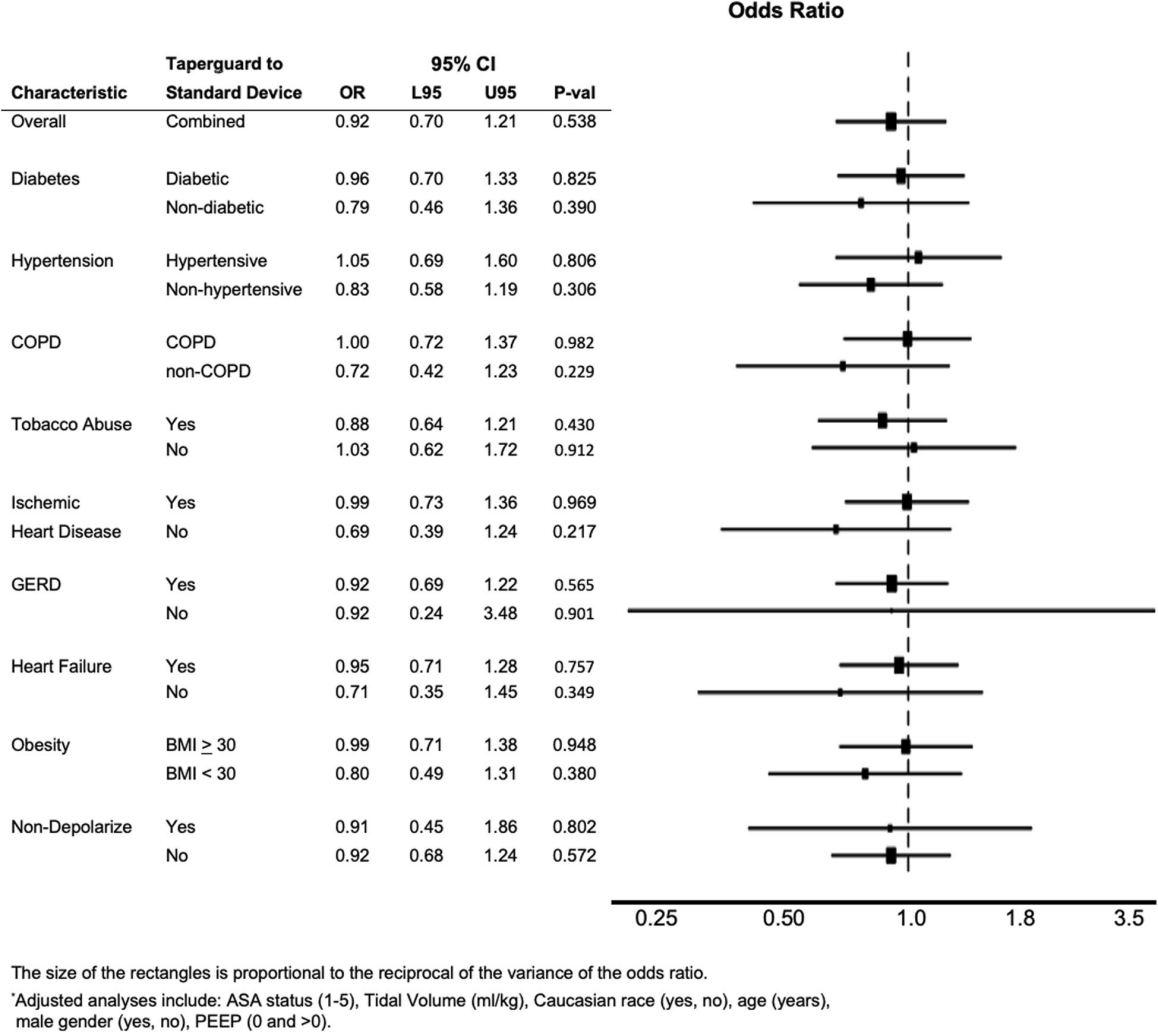
Odds Ratios of VAP comparing the TaperGuard™ ETT with the standard ETT, among population subgroups

The results of our investigation of possible effect modification of the relationship between ETT cohort and postoperative pneumonia are shown in Fig. 2. We did not find evidence that any of the disease or subclinical disease groups modified the association between ETT cohort and postoperative pneumonia (all *p*-values > 0.20).

## Discussion

To our knowledge, this study represents the largest evaluation of the effectiveness of TaperGuard™ ETTs for the prevention of postoperative pneumonia in a large, heterogeneous, surgical population. We found no difference in the odds of developing postoperative pneumonia during the use of the TaperGuard™ ETT relative to the use of the standard ETT. We also found no differences in postoperative pneumonia between the two cohorts among higher pneumonia risk subgroups. Considering previous work, these findings substantiate previous findings that the TaperGuard™ ETT does not have a role in preventing postoperative pneumonia.

The efficacy of the TaperGuard™ ETT in reducing incidence of VAP has been previously investigated. Bowton et al. conducted an observational, two-period study of ICU patients and found no reduction in VAP rate with hospital and community-wide implementation of the TaperGuard™ ETT [15]. Similar to ours, the VAP bundle adherence was high, resulting in relatively low incidence of VAP, suggesting that the study may not have had adequate power to detect a difference. Bowton et al. defined VAP based on National Healthcare Safety Network criteria [17], because all of their patients had ICU admission. We evaluated the TaperGuard™ ETT in a less selected patient population, who may have received tracheal intubation and mechanical ventilation only during the perioperative period. Due to the broader population, our ability to screen for and detect pneumonia events was limited to data collection performed on the least critically ill inpatients, meaning we were unable to apply National Healthcare Safety Network criteria. The highest quality data source for determination of postoperative pneumonia in our sample was hospital discharge ICD-9 codes for bacterial, fungal, and ventilator-associated pneumonia. It is possible that ICD-9 codes were not coded accurately, and the diagnostic processes leading to the application of codes were not available for us to review in aggregate. We also may have not captured a subset of hospitalized patients who were discharged from the hospital prior to onset of symptoms of pneumonia. However, our sample size was sufficiently large and there is no reason to suspect a difference in the duration or quality of postoperative mechanical ventilation practices or discharge behaviors between the two study periods. Due to the large sample, the estimates are precise, and the associated statistical tests have sufficiently high statistical power. Assuming an incidence of postoperative pneumonia of 1.8% for the standard ETT, the study would exceed 80% power to detect a reduction to 1.2% in the incidence of VAP in the TaperGuard™ ETT. At out institution, we also utilize a VAP prevention bundle as part of the standard of care for all intubated patients, and the components of the bundle did not change over the entire study period. However, it remains possible that due to unmeasured improvements in healthcare over time or unrecognized changes in care delivery between the cohorts, there are unmeasured confounding differences between the cohorts.

The rationale of the tapered cuff design of the TaperGuard™ ETT is to create a more complete seal around the tracheal wall, thus reducing micro-channels that allow the leakage of supraglottic secretions below the cuff. Multiple small laboratory studies have evaluated the degree to which the TaperGuard™ ETT reduces leakage of fluid around an inflated cuff in an experimental tracheal model. Most have found that in static conditions, the tapered cuff reduces the passage of fluid below the cuff [8–14]. Experimental studies that more closely mimic the physiologic conditions of tracheal intubation and ventilation suggest less sustained effects. One study found that the ability of TaperGuard™ ETT to reduce fluid leakage decreased significantly with PEEP < 10 cm H_2_O and with intubation times longer than 60 min [10]. Another study using microbial suspensions of *Staphylococcus*, *Pseudomonas* and *Candida* above the cuff revealed that the TaperGuard™ ETT failed to prevent inoculation of the space below the cuff [11]. The degree of inoculation also varied based on the diameter of the experimental tracheal model used, and the volume of fluid leakage.

The available evidence suggests that the TaperGuard™ ETT reduces measurable fluid leakage but does not prevent the passage of fluid or microorganisms, especially not over a broad set of conditions or for clinically relevant time periods. Measurement of microaspiration in vivo has not been thoroughly evaluated [14–16, 18]. In one study, 60 patients scheduled for lumbar spine surgery were intubated and equally randomized to receive a standard ETT or the TaperGuard™ ETT0 [18]. Dye was instilled into the supraglottic space, and bronchoscopy was performed to assess the degree of dye descent along the cuff up to 2 h. The TaperGuard™ ETT allowed dye leakage up to the second third of the cuff, but none into the subglottis. It is conceivable that the reduced but incomplete degree of protection from microaspiration of the tapered cuff design could contribute to the lack of effect on VAP prevention observed in our study. To our knowledge, the only other randomized trial that compared the standard ETT to the TaperGuard™ ETT was conducted in 109 high-risk patients undergoing major vascular surgery [16]. All of their patients were transferred to the ICU and screened daily for clinical suspicion of VAP until 5 days post extubation, up to 28 days. Tracheal aspirates were also sampled for pepsin and amylase to measure microaspiration. While the incidence of pneumonia was relatively high (42–44%) in that study, there was no difference in the incidence of VAP between groups. Additionally, there were no differences in amylase and pepsin concentrations from tracheal aspirates on postoperative days 1 and 2. Taken together, these findings suggest that the TaperGuard™ ETT does not appear to reliably prevent microaspiration or VAP even in high-risk groups.

## Conclusion

The current study contributes to a growing body of literature suggesting that the TaperGuard™ ETT is not an effective device for broad implementation in surgical patients to reduce the risk of VAP in the perioperative setting. However, cuff shape is only one component of endotracheal ETT design modifications, and there are other possible configurations or material of cuffed ETTs that may be more effective. The inclusion of subglottic suctioning, or a polyurethane rather than polyvinylchloride cuff, are features that may be more important for VAP reduction, especially in high-risk patient groups. Based on collective evidence, TaperGuard™ ETT does not appear to prevent postoperative pneumonia. Utilization of this specialized ETT for the prevention of postoperative pneumonia in an unselected surgical patient population is not warranted.

## Data Availability

The datasets used and/or analyzed during the current study are available from the corresponding author on reasonable request.

## Abbreviations

VAP: Ventilator associated pneumonia
ETT: Endotracheal tube
OR: Odds ratio
OHSU: Oregon Health & Science University
NSQIP: National Surgical Quality Improvement Project
ASA: American Society of Anesthesiologists
LOS: Length of stay
PEEP: Positive end-expiratory pressure
